# An Omnidirectional Polarization Detector Based on a Metamaterial Absorber

**DOI:** 10.3390/s16081153

**Published:** 2016-07-23

**Authors:** Binzhen Zhang, Yong Zhang, Junping Duan, Wendong Zhang, Wanjun Wang

**Affiliations:** 1 Science and Technology on Electronic Test & Measurement Laboratory, North University of China, Taiyuan 030051, China; zhangbinzhen@nuc.edu.cn (B.Z.); duanjunping@nuc.edu.cn (J.D.); wdzhang@nuc.edu.cn (W.Z.); 2 School of Instrument and Electronics, North University of China, Taiyuan 030051, China; 3 Department of Mechanical Engineering, Louisiana State University, Baton Rouge, LA 70803, USA; wang@lsu.edu

**Keywords:** omnidirectional, polarization, metamaterial, absorber, detector

## Abstract

The theory, design, simulation, fabrication, and performance of an omnidirectional polarization detector (PD) with two resonances located in the X and Ka ranges based on a metamaterial absorber (MMA) are presented in this paper. The sandwich structure of PD is composed of 0.1 μm periodic “I” shaped patches on the metasurface, a dielectric of 200 μm FR-4 on the interlayer, and a 0.3 μm copper film on the substrate. PD absorptivity is first used to reflect and describe the polarization of the incident wave. The numerical results, derived from the standard full wave finite integration technology (FIT) of CST 2015, indicates that the designed PD shows polarization sensitivity at all incidence angles. The effects on absorptivity produced by the incidence angles, polarization angles, and materials are investigated. The amplitude of absorptivity change caused by polarization reaches 99.802%. A laser ablation process is adopted to prepare the designed PD on a FR-4 board coated with copper on the double plane with a thickness that was 1/93 and 1/48 of wavelength at a resonance frequency of 16.055 GHz and 30.9 GHz, respectively. The sample test results verify the designed PD excellent detectability on the polarization of the incident waves. The proposed PD, which greatly enriches the applications of metamaterials in bolometers, thermal images, stealth materials, microstructure measurements, and electromagnetic devices, is easy to mass produce and market because of its strong detectability, ultrathin thickness, effective cost, and convenient process.

## 1. Introduction

Polarization is one of the significant characteristics of electromagnetic waves that plays a vital role in many domains, such as polarization imaging, confidential communications, heterodyne detection, titers, navigation, and quantum computers. However, the lack of convenient PD with high-performance limits the exploitation of polarization. Polarization analyzers based on conventional polarization detection methods, which include polarization interferometry or modulation detection [1,2], four probes distribution detection [3], and polarization amplitude detection [4], are difficult to adopt because of their complicated mechanical structure, and immense data processing. Electric vector, Jones vector, Stokes vector, and Poincare sphere, which are intricate and elusive, are usually adopted to describe the polarization of the incident waves [5,6]. Tong et al. attempted to utilize the polarization of electromagnetic waves to detect the gravitational waves [7]. The Laser Interferometer Gravitational Wave Observatory (LIGO) remains unable to detect relic gravitational waves (RGWs) by two orders of magnitude even though it has achieved its design sensitivity [8]. Leszek et al. successfully input the pending test waves into the Sagnac loop [1]. Polarization was extracted from the detection of the current signal in two channels and complicated aftertreatment. However, the system applied phase modulation, which requires a rigorous surrounding to control stray light, making this method unsuitable for detecting the polarization of high-speed and real-time signals because of its difficult structure adjustment, environmental sensitivity, and tedious calculation process. Azzam acquired the Stokes vector, which is adopted to reflect and describe polarization, by calculating the currents value of four photodiodes that were placed around the optical fiber, which can be detected directly and conveniently [3]. Nevertheless, this method has considerable limitations in expansion and understanding on account of the extremely stable work environment and accurately gauged photodiodes. Grimberg et al. proposed a neotype electromagnetic sensor composed of a metamaterial lens and conical Swiss rolls that can manipulate evanescent waves existent in near field or in slits of metallic strip gratings, in the space between carbon fibers in carbon fiber reinforced plastic (CFRP) composites, as well as in cracks with microscopic features of conductive bodies [9,10,11]. Nowadays, researchers usually adopt a massive and precise system to detect and analyze the polarization of electromagnetic waves. However, these existing detection and analysis systems encounter difficulties in a lack of precise understanding, complicated mechanical structures, immense data processing needs, and environmental sensitivity which limit their usability and marketability.

Since the first theoretical [12] and experimental [13] demonstrations of left-handed materials (LHMs), scientific research into metamaterials has developed rapidly and enormously. Metamaterials are a kind of artificial composite material with periodic arrangement of subwavelength metal resonators. The unique properties of metamaterials, including negative refraction [14], anomalous Cerenkov radiation [15], abnormal Doppler shift [16], and reverse Goos-Hanchen shift [17] increase their potential utility in various fields, such as bolometers, thermal imaging, stealth materials, perfect lenses, microstructure measurements, communication devices, and electromagnetic devices that contain cloaks, absorbers, collectors, rotators, splitters, and detectors [18,19,20].

Incidence insensitive PDs are urgently needed because of advancements in microwave science. Currently, research into PDs based on MMAs is scarce. Most proposed MMAs with resonators that possess symmetric microstructures are polarization insensitive [21]. Thus, we designed a smaller but complicated PD based on an omnidirectional MMA in this paper. Incident waves with various polarizations generate different absorptivities. Then, we can acquire the polarizations of the incident waves according to the frequency and absorptivity peaks in the measured absorptivity curve. We may just adopt a detector based on a MMA to detect the polarization in the future, rather than a huge and complicated system with conventional approaches. The novel omnidirectional PD based on a MMA that consisted of 0.1 μm periodic “I” shaped patches on the metasurface, a dielectric of 200 μm FR-4 on the interlayer, and a 0.3 μm copper film on the substrate is demonstrated in this paper. The response frequency of a PD prepared on FR-4 is lower than 30 GHz because of the low precision of the conventional etching process, with a typical value of 80 μm. The PD with responses at higher frequency is usually processed by photolithography on polymer which is tedious and difficult to adopt in large-scale production. Therefore, laser ablation was adopted to prepare the designed PD on a FR-4 board coated with copper on the double plane with a thickness that is 1/93 and 1/48 of wavelength at the resonance frequencies of 16.055 GHz and 30.9 GHz, respectively. The PD absorptivity calculated by *A*(ω) = 1 − |*S*_11_|^2^ − |*S*_21_|^2^ is adopted to reflect the variations of the scattering parameters caused by the changes of the polarization. The response can fall in the wireless communications band (2.4 GHz) and THz band (100 GHz–10,000 GHz) by adjusting the resonator size. The number of response bands can be modulated by increasing or decreasing the number of resonators [22,23,24]. The proposed PD possesses ample potential in scientific research (e.g., polarization optics, and electromagnetic coupling) and engineering applications (e.g., polarized glasses, confidential communication, biochip detection, photomemory, bolometers, thermal imaging, microstructure measurements) because of its strong detectability, ultrathin thickness, low cost, and convenient manufacturing process.

## 2. Theory, Structure Design and Simulations

### 2.1. Theoretical Analysis and Structure Design

When electromagnetic waves (such as light) traverse a metamaterial, the resonance of the electric and magnetic fields can activate the electrons in atoms or molecules. As a result, the transmission properties will be changed as the material consumes the energy of the electromagnetic waves. Natural materials comprise microatoms whose polarization and magnetization characteristics determine the material electromagnetic response. Complex permittivity $\varepsilon \left(\omega \right)=\varepsilon^{\prime }+i\varepsilon^{\prime \prime }$ and complex permeability $\mu \left(\omega \right)=\mu^{\prime }+i\mu^{\prime \prime }$ are adopted to describe the acuteness degree of motion. ε′ is a parameter that describes the degree of polarization, whereas μ′ describes the degree of magnetization. ε′′ and μ′′ are the basic characterization parameters that describe the electromagnetic losses in a medium.

Electromagnetic waves with low frequency, that incide on the metasurface of the designed PD, will excite the electrons over a wide range and make the electrons absorb the most energy. The small induced currents in the corner of the “I” patches lead to a large *R*(ω). The movement range of electrons will gradually become smaller, and the induced currents in the corner will gradually become larger with the increase of the incident waves’ frequency. When the frequency of the incident waves reaches a certain value, electrons in the two sides of “I” patches fleetly move back and forth with the actuation of electric field vector of the incidence waves. Then, the induced currents reach the maximum value. The incident electromagnetic waves are absolutely coupled into the dielectric layer at resonance points, and *R*(ω) approaches zero. When the incident wave frequency rises continually, the movement range of electrons will become smaller, the strong induced currents will be divided into several segments, so *R*(ω) will begin to increase. The metasurface of the proposed PD can serve as a frequency selective surface (FSS) with a band pass filter function.

For a sandwich structured PD that contains the metasurface, a dielectric layer, and a metal substrate, $A\left(\omega \right)=1-R\left(\omega \right)-T\left(\omega \right)=1-{\left|S_{11}\right|}^{2}-{\left|S_{21}\right|}^{2}$ can be applied to calculate the absorptivity. $R\left(\omega \right)={\left|S_{11}\right|}^{2}$ is reflectivity and $T\left(\omega \right)={\left|S_{21}\right|}^{2}$ is transmittivity. Precise control of *n*(ω) and the PD input impedance *Z*(ω) is necessary for realizing a high absorptivity. Electromagnetic metamaterials are prime candidates for this task because they can be designed to couple to electric and magnetic components of incident waves. This enables precise tuning of the complex, and frequency-dependent ε(ω) and μ(ω). The index *n*(ω) and the PD impedance *Z*(ω), which are determined by the metamaterial parameters and electromagnetic properties of the incoming waves, are preliminarily expressed as $n\left(\omega \right)=\;\sqrt{\mu \left(\omega \right)\varepsilon \left(\omega \right)}$ and $Z\left(\omega \right)=\sqrt{\mu \left(\omega \right)/\varepsilon \left(\omega \right)}$, respectively. *R*(ω), which can by calculated from the formulation (1), is controlled by the incidence angle θ, the matching degree of *Z*(ω) and *Z*_0_.

(1) $$R\left(\omega \right)={\left|S_{11}\right|}^{2}=\frac{{\left[\left(Re\left\{Z\left(\omega \right)\right\}-Z_{0}cos\theta \right)\right]}^{2}+{\left[Im\left\{Z\left(\omega \right)\right\}\right]}^{2}}{{\left[\left(Re\left\{Z\left(\omega \right)\right\}+Z_{0}cos\theta \right)\right]}^{2}+{\left[Im\left\{Z\left(\omega \right)\right\}\right]}^{2}}$$

where $Z_{0}=\sqrt{\mu_{0}/\varepsilon_{0}}=337\;\Omega$ is a constant that denotes the wave impedance of free space [25]. The effect of the surface resistance of the FSS and dielectric characteristics on the input impedance of the metamaterial is discussed by means of a circuital model. *Z*(ω) can be equivalent to a parallel connection between dielectric impedance $Z_{d}\left(\Omega \right)$ and FSS impedance $Z_{fss}\left(\Omega \right)$ according to Equation (2):

(2) $$\frac{1}{Z_{\omega }}=\frac{1}{Z_{d}}+\frac{1}{Z_{\text{fss}}}$$

The expression of *Z*_d_ with TE and TM polarization at oblique incidence well reads with the relation (3):

(3) $$Z_{d}=iZ_{d_{0}}^{TE,TM}\left[k_{0}h_{d}\sqrt{\varepsilon^{\prime }+i\varepsilon^{\prime \prime }-sin^{2}\left(\theta \right)}\right]$$

where $Z_{d0}^{TE}=\left({\omega \mu \mu }_{0}\right)/\beta ,\;Z_{d0}^{TM}=\beta /\left({\omega \mu \mu }_{0}\right)$ are the characteristic impedances of the dielectric for TE and TM polarization [26]. $\beta =\sqrt{k_{0}^{2}-k_{t}^{2}}=k_{0}\sqrt{1-\sin^{2}\left(\theta \right)}$ is the propagation constant and *k_t_* = *k*_0_sin(θ) is the transverse wavenumber. θ represents the incidence angle with respect to the vertical incidence. The real and the imaginary part of *Z*_d_ can be well calculated from Equations (4) and (5), respectively:

(4) $$Re\left\{Z_{d}\right\}=\frac{Z_{0}}{\sqrt{\varepsilon^{\prime }}}\left\{\frac{\varepsilon^{\prime \prime }}{2\varepsilon^{\prime }}tg\left(k_{0}h_{d}\sqrt{\varepsilon^{\prime }}\right)-\left(k_{0}h_{d}\frac{\varepsilon^{\prime \prime }}{2\sqrt{\varepsilon^{\prime }}}\right)\left[1+tg^{2}\left(k_{0}h_{d}\frac{\varepsilon^{\prime \prime }}{2\sqrt{\varepsilon^{\prime }}}\right)\right]\right\}$$

(5) $$Im\left\{Z_{d}\right\}=i\frac{Z_{0}}{\sqrt{\varepsilon^{\prime }}}\left[tg\left(k_{0}h_{d}\sqrt{\varepsilon^{\prime }}\right)\right]$$

where *h*_d_ represents the dielectric thickness. After some mathematical and analytical manipulations with the help of [27], Im{*Z*_d_} with TE and TM polarization at oblique incidence can be well approximated by the following relations:

(6) $$Im{\left\{Z_{d}\right\}}^{TE}\approx i\omega \mu_{0}h_{d}$$

(7) $$Im{\left\{Z_{d}\right\}}^{TM}\approx i\frac{\varepsilon^{\prime }-sin^{2}\left(\theta \right)}{\varepsilon^{\prime }}\omega \mu_{0}d\approx i\cos\left(\theta \right)\omega \mu_{0}h_{d}$$

The formulas (6) and (7) show that the reactance of Im*^TE^*{*Z*_d_} is almost unchanged with the θ. However, the increase of θ leads to a decrease of Im*^TM^*{*Z*_d_}. The adoption of the dielectric with a higher ε′ would increase the sensitivity of θ, however, a defective absorption would be obtained for very oblique incidence such as θ = 80°. The perfect absorption with higher θ for TE polarization is instead more difficult to reach.

*Z*_fss_, which stands for the impedance of the lossy FSS, can be represented through a series RLC circuit, and it can be extracted from the following formulas:

(8) $$Z_{FSS}=R_{o}+R_{d}-iX$$

(9) $$R_{o}=\frac{1}{\delta \sigma }{\left(\frac{2p}{l_{1}+l_{3}}\right)}^{2}$$

(10) $$R_{d}=\frac{-\varepsilon^{\prime \prime }}{\omega \left(1+\varepsilon^{\prime }\right)\left[C-\frac{2p\varepsilon_{0}}{\pi }log\left(1-e^{\frac{-4\pi h_{d}}{p}}\right)\right]}$$

(11) $$X=\frac{1-LC\omega^{2}}{\omega C}$$

where *p* is the length of periodic array, *l*_1_ and *l*_2_ stand for the structure dimensions of a “I” patch. *Z*_fss_, as previously remarked in (8), is composed by three loss terms, an ohmic resistor, *R*_o_, a dielectric resistor, *R*_d_, and *X* [28]. The shape and size of FSS decide the values of *L* and *C*. It is shown that resonators with high capacitive elements, keeping ε unchanged, need a dielectric with higher losses to achieve perfect absorption, but they lead to the maximization of the full width at half maximum (FWHM). It is shown that the optimum value of *Z*_fss_ is affected both by *h*_d_, ε, and, shape and size of resonator in FSS. The FSS with a small resonator filling factor are characterized by high ohmic losses due to high surface currents. A patch type configuration allows minimizing the ohmic losses and maximizing the dielectric ones, but as already pointed out, it requires a high tangent loss of the dielectric or an adequately thin dielectric to obtain strong resonance and perfect absorption. The adoption of abundant lumped resistors for introducing ohmic losses in the metasurface leads to complex and expensive structures because of the cost of high frequency resistors and complexity of the conventional soldering. An attractive alternative is printing the periodic metal patterns with a proper surface resistance.

Synthesizing the above analysis, the input impedance can be expressed in detail as follows:

(12) $$Z_{\omega }=\frac{\left(R_{e}\left\{Z_{d}\right\}R-I_{m}\left\{Z_{d}\right\}X\right)\left(R_{e}\left\{Z_{d}\right\}+R\right)+\left(I_{m}\left\{Z_{d}\right\}R-R_{e}\left\{Z_{d}\right\}X\right)\left(I_{m}\left\{Z_{d}\right\}+X\right)}{{\left(R_{e}\left\{Z_{d}\right\}+R\right)}^{2}+{\left(I_{m}\left\{Z_{d}\right\}+X\right)}^{2}}$$

where *R* = *R*_0_ + *R*_d_. After some analytical manipulations [29], the real and the imaginary part of the input impedance *Z*ω can be derived as the following formulas:

(13) $$Re\left\{Z_{\omega }\right\}=\frac{RZ_{d}^{2}}{{\left(\frac{1-LC\omega^{2}}{\omega C}-Z_{d}\right)}^{2}+R^{2}}$$

(14) $$Im\left\{Z_{\omega }\right\}=\frac{\left(\frac{Z_{d}}{\omega C}-\omega LZ_{d}\right)\left(\frac{1-LC\omega^{2}}{\omega C}-Z_{d}\right)+R^{2}Z_{d}}{{\left(\frac{1-LC\omega^{2}}{\omega C}-Z_{d}\right)}^{2}+R^{2}}$$

The increase of the loss components in the dielectric causes a reduction of Re{*Z*ω}. A reduced loss leads to the Re{*Z*ω} being higher than the free space impedance while a too lossy dielectric would reduce the absorptivity since Re{*Z*ω} becomes smaller than the free impedance. A moderate red shift of resonance frequency will occur with the increase of *h*_d_ that leads to the increase of the dielectric inductance.

Reflectivity becomes zero when the PD reaches perfect impedance-matched (*Z*(ω) = *Z*_0_), which can be achieved by designing appropriate microstructures and arrangements. The incident electromagnetic wave is absolutely coupled into the dielectric layer at resonance points. The middle dielectric layer is responsible for consuming electromagnetic energy. Positive transfer coefficient *S*_21_ was determined to be dependent on the complex index of refraction *n*(ω) = *n*′ + i*n*′′ and the thickness of dielectric *h*_d_ as relation (15):

(15) $$S_{21}^{-1}=e^{-ikh_{d}}\left[\sin\left(nkh_{d}\right)-icos\left(nkh_{d}\right)\right]=e^{-i\left(n^{\prime }-1\right)kh_{d}}e^{n^{\prime \prime }kh_{d}}$$

where *k* = ω/*c* and c is the speed of light in vacuum. Thus, transmissivity *T*(ω) = |*S*_21_|^2^ = *e*^−2*n*′′*kh*^. The combined dielectric and magnetic losses in the system are characterized by *n*′′ and *h*. Therefore, when *n*′′ or h approaches infinity, *T*(ω) approaches zero [30]. The metal base guarantees that the incident wave does not cross the substrate when the thickness of metal substrate is greater than skin depth which results in transmittivity $T\left(\omega \right)={\left|S_{21}\right|}^{2}=0$ [31].

**Figure 1:**
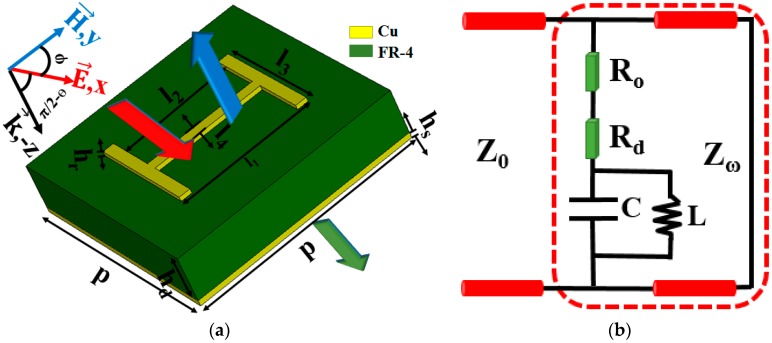
The schematic diagram of PD (**a**) and its equivalent circuit (**b**).

The arrangement and single resonator are depicted in Figure 1. The electric fields, which are parallel to the direction of the “I” shaped patch, oscillate the electrons bounded in an infinitely long straight wire and generate induced currents on the metasurface. Therefore, a portion of the incident waves’ energy can be consumed by the oscillating electrons while the surplus energy penetrates the metasurface and diffuses continually. When the plane, where the wire is located, is rotated by 90 degrees while keeping the electromagnetic properties of the incident waves unchanged, absorption and consumption will decline dramatically. A full wave electromagnetic (EM) simulation is performed based on the standard finite integration technology (FIT) in CST Microwave Studio 2015. Periodic boundary conditions are used in the *x* and *y* directions, and the excitation source is a Floquet port with diverse incidence angles and polarizations. The project parameters are presented in Table 1. Thus, absorptivity can be calculated by utilizing the equation $A\left(\omega \right)=1-R\left(\omega \right)-T\left(\omega \right)=1-{\left|S_{11}\right|}^{2}-{\left|S_{21}\right|}^{2}$.

### 2.2. Simulation Analysis

**Figure 2:**
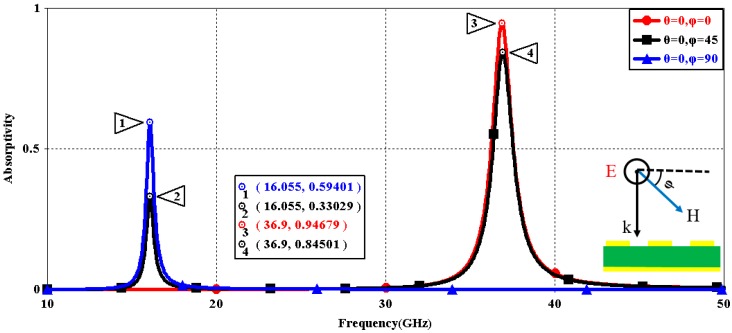
Simulated absorptivity with different polarization angles (TE, θ = 0°).

**Figure 3:**
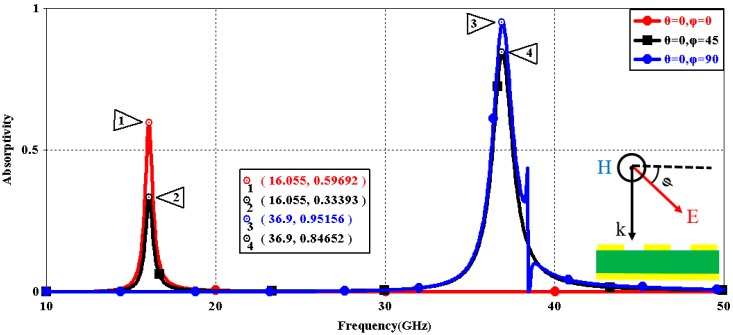
Simulated absorptivity with different polarization angles (TM, θ = 0°).

**Figure 4:**
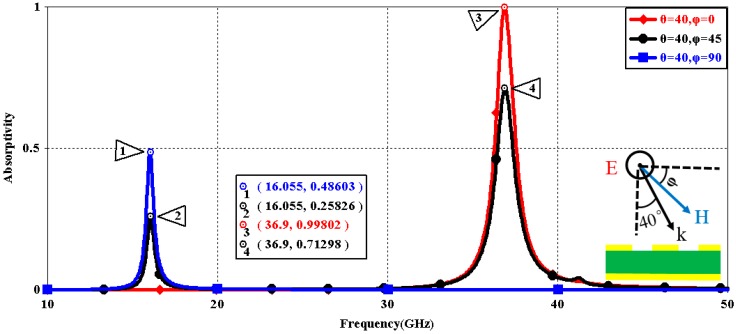
Simulated absorptivity with different polarization angles (TE, θ = 40°).

**Figure 5:**
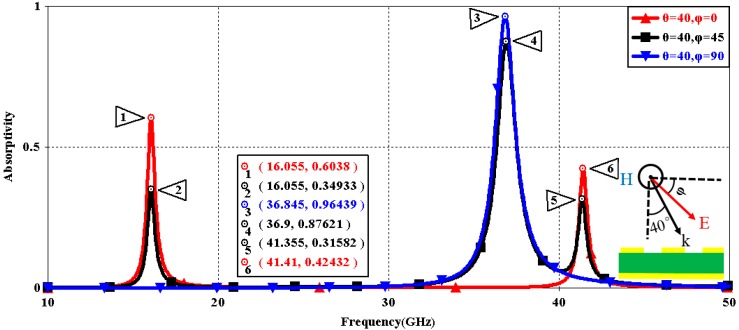
Simulated absorptivity with different polarization angles (TM, θ = 40°).

**Figure 6:**
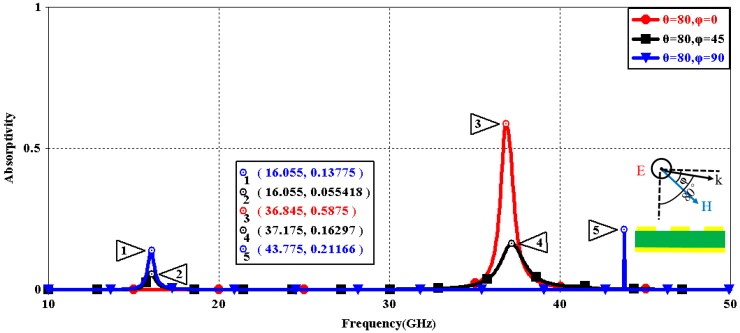
Simulated absorptivity with different polarization angles (TE, θ = 80°).

**Figure 7:**
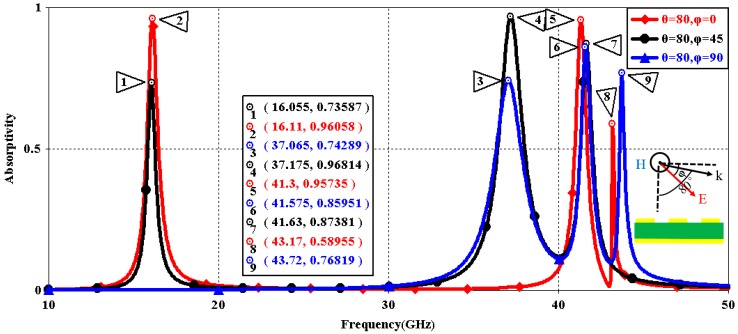
Simulated absorptivity with different polarization angles (TM, θ = 80°).

**Figure 8:**
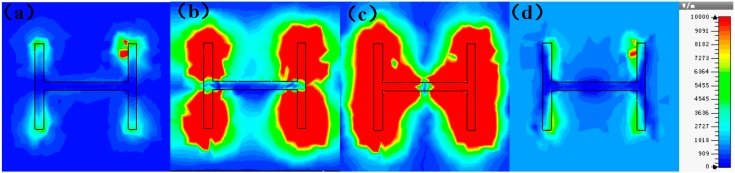
The distributions of electric fields in resonance points. (**a**) TE, θ = 0°, ϕ = 0°, 16.05 GHz; (**b**) TM, θ = 0°, ϕ = 0°, 16.05 GHz; (**c**) TE, θ = 0°, ϕ = 0°, 36.9 GHz; (**d**) TM, θ = 0°, ϕ = 0°, 36.9 GHz.

**Figure 9:**
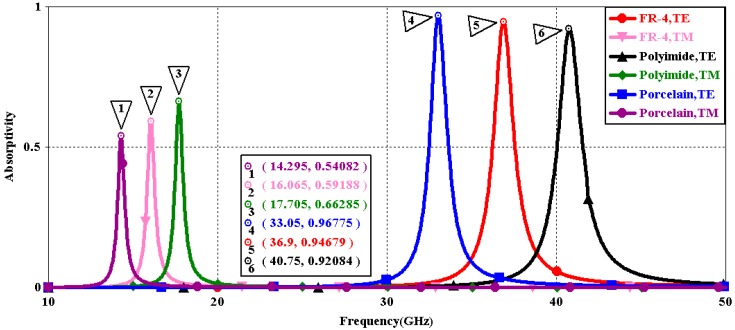
Simulated absorptivity at different ε_media (θ = 0°, ϕ = 0°).

**Figure 10:**
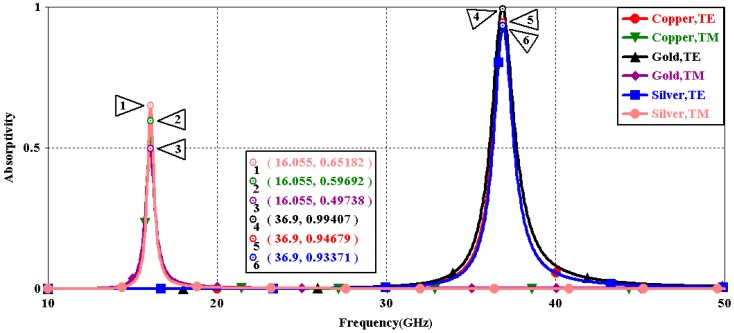
Simulated absorptivity at different σ_meta (θ = 0°, ϕ = 0°).

We first explored the connection between incident wave polarization and absorptivity with various incidence angles. The simulated results are shown in Figure 2, Figure 3, Figure 4, Figure 5, Figure 6 and Figure 7. Figure 2 indicates that the transverse electromagnetic (TEM) waves with vertical incidence generate an absorptivity of 0.94679 at 36.9 GHz, which is the strongest resonance point. When the angle of incidence remains the same (θ = 0°), and the polarizing angle increases in 45° steps (ϕ = 0°, 45°, 90°), Figure 2 shows that the increased polarization angle of the transverse electric (TE) waves causes a decline of absorptivity peak in the high band and the appearance of an absorptivity peak at 16.055 GHz, which is located in the low band. In other words, the absorptivity peak is directly proportional to the polarization angle of the TE waves in the low band, whereas the proportion reverses in the high band. Figure 3 reveals the inverse of the proportional relationship between the absorptivity peak and the polarization angle when the incidence waves become transverse magnetic (TM) waves. The elaborative comparison of the two illustrations reveals that there is no shift of resonance points when the polarization state of the incidence waves changes. Figure 4 and Figure 5 show that when the incidence angle is increased to 40° (θ = 40°), the proportionality relationship between the absorptivity peak and the polarization angle remains the same in both the high band and the low band for all polarization states. The comparison of Figure 4 and Figure 2 reveals that the absorptivity peaks decline in the resonance points, except for the case of θ = 40°, ϕ = 0°. The contrast of Figure 5 and Figure 3 shows that the decreased absorptivity peaks increase. In the case of TM, θ = 40°, ϕ = 45°, 90°, Figure 6 and Figure 7 shows that a new resonance is detected near 41.4 GHz. Absorptivity decreases dramatically when θ is increased to 80° during the stimulation of TE waves, while TM waves exhibit stronger resonance and higher absorptivity. The new resonance point locates in 43.755 GHz during the stimulation of TE, θ = 80°, ϕ = 90°, whereas the TM waves cause two fresh resonances near 41.6 GHz and 43.5 GHz. The absorption properties and mechanism of the designed PD are further analyzed by demonstrating the electric fields distributions of the resonator surface in Figure 8. Strong resonance occurs in the corners of periodic “I” shaped patches. In the case of θ = 40°, ϕ = 0°, the TE waves cause a powerful resonance at 36.9 GHz, whereas the TM waves cause a forceful resonance at 16.055 GHz. The polarization of the incident waves can be achieved from the analysis on the absorptivity of PD with the help of Table 2. Then, we investigate the effects on absorptivity produced by electromagnetic properties of the dielectric. Figure 9 shows the transformation of absorptivity and resonance points of PD when the middle dielectric is porcelain, FR-4, and polyimide with a permittivity of 5.7, 4.4, and 3.5, respectively. The image indicates that the increase of dielectric permittivity triggers a red shift of the resonance frequency, an increase of absorptivity in the high band, and a reduction of absorptivity in the low band. The relevance help concerned individuals to select a suitable dielectric to project the appropriate PD for special circumstances. Finally, in order to study the relationship between the conductivity of metallic material and the absorbing performance of PD, we defined the metal as gold, copper, and silver with conductivity of 4.5e7 S/m, 5.8e7 S/m, and 6.3e7 S/m, respectively. As shown in Figure 10, the metal conductivity mainly affects the absorptivity peak value rather than the resonance frequency.

## 3. Processing and Testing

**Figure 11:**
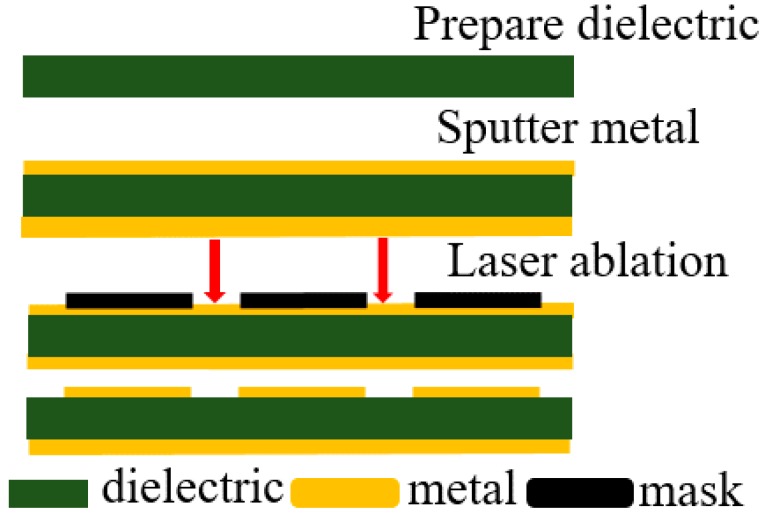
Flow chart of process.

**Figure 12:**
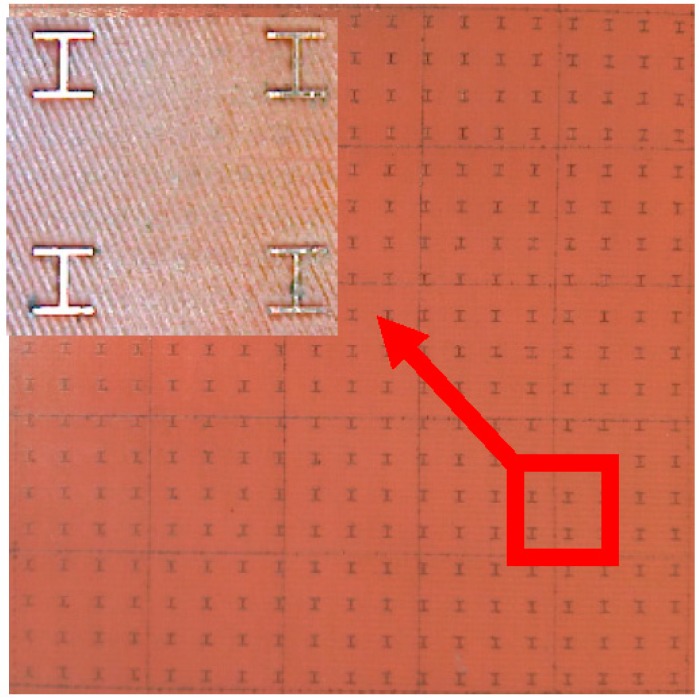
Optical microscopy image of a portion of the fabricated PD and the inset shows the enlarged unit cell.

To verify the validity of the simulation results, the physical dimensions and material parameters of the manufactured MMA were determined by the optimized analysis presented in Section 2. A type JTRC-550 magnetron sputtering machine (Jiangtai vacuum coating technology Co., Ltd, Chengdu, China) was employed to sputter 0.1 μm copper and 0.3 μm copper on the 40 cm × 40 cm × 200 μm FR-4 board’s front and back, respectively. Then, the redundant copper film on the front was removed using LPKF ProtoLaser U3 laser burning equipment (LPKF Laser & Electronics AG, Garbsen, Germany). The left copper film’s structure is shown in Figure 1. The flow chart of the process is demonstrated in Figure 11. Figure 12 shows the prepared sample and its local amplifying structure.

**Figure 13:**
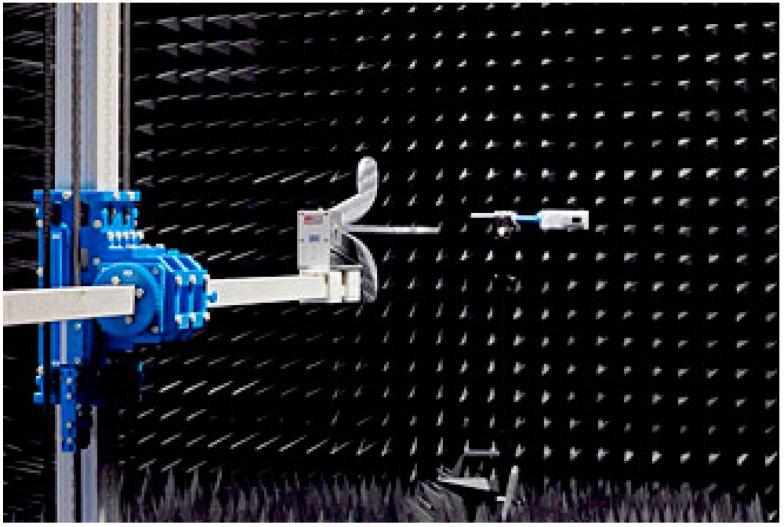
The anechoic chamber layout.

**Figure 14:**
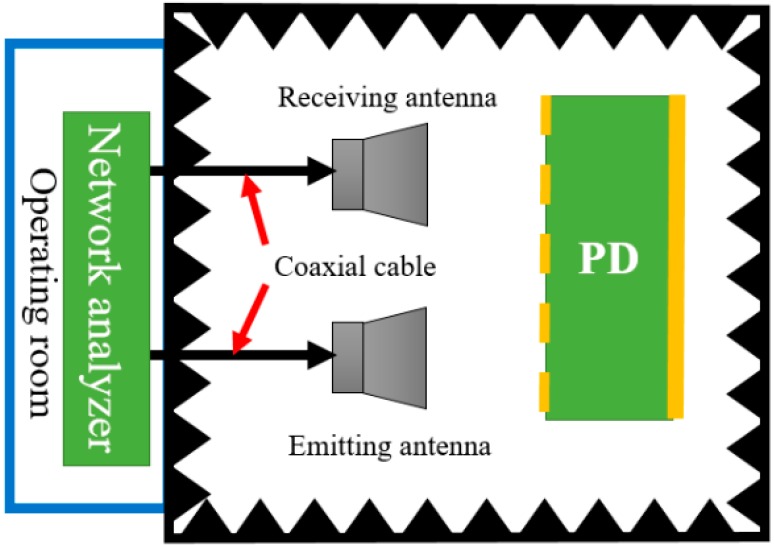
Schematic of the measurement.

**Figure 15:**
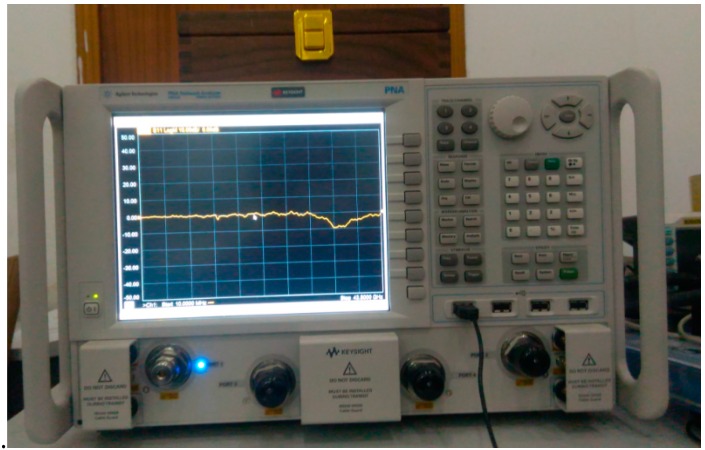
Photograph of the Agilent N5224A.

**Figure 16:**
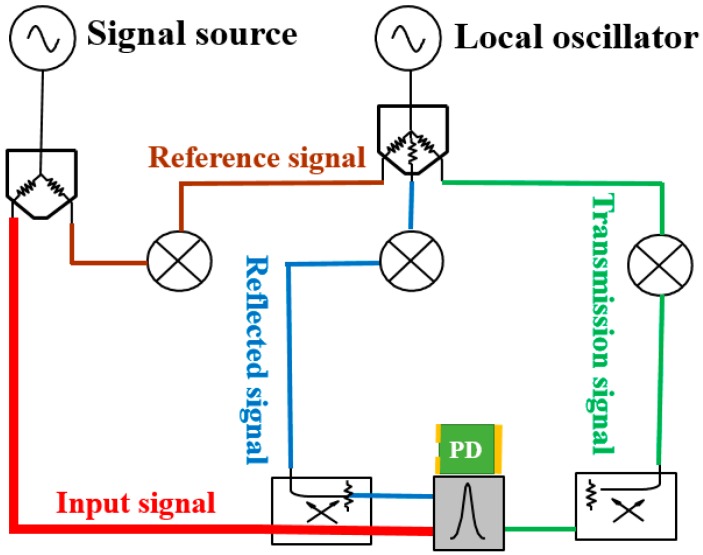
Graph of the signal flow.

We adopt the free-space reflection method in an anechoic chamber to test the scattering parameters of the sample. The scene and schematic of the free-space reflection method are shown as Figure 13 and Figure 14. In order to ensure that the electromagnetic waves which incide upon the sample surface are plane waves d, the distance between the antenna and the sample, should meet the far field condition d > (2D^2^)/λ, where λ is the wavelength of the incident waves, and D is the diagonal length of the antenna. In addition, in order to eliminate the sample edge scattering effects which will affect the measurement precision, the size of the test sample should be at least more than two times the maximum working wavelength. The signal source of an Agilent N5224A network analyzer Agilent Technologies, Santa Clara, CA, USA) (shown in Figure 15) with frequency expansion module of 500 GHz is responsible for generating the excitation signal that meets the requirements of frequency and power. The excitation signal is divided into two signals by a power splitter. One of them is served as input signal marked red line in Figure 16, and another one as reference signal marked with a brown line. Then, the input signal is radiated by an emitting antenna. Radiant electromagnetic waves incident upon the metasurface of the prepared PD. A part of the incident waves’ energy will be reflected, the surplus energy will be coupled into the metasurface, and then, diffuse continually in the dielectric.

**Figure 17:**
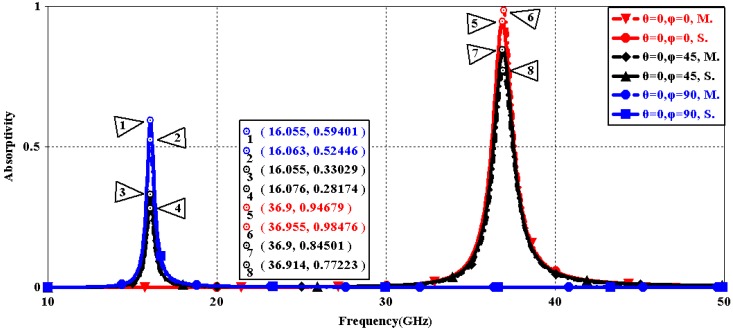
The contrast of simulation and measurement results (TE, θ = 0°).

**Figure 18:**
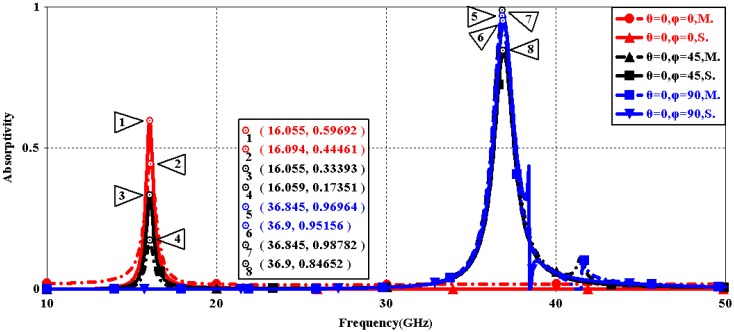
The contrast of simulation and measurement results (TM, θ = 0°).

The contrastive diagrams of the test and simulation results with a vertical incidence and different polarization angles of the TE and TM waves are presented in Figure 17 and Figure 18. The contradictory results indicate that slight resonance point and absorptivity shifts occurred. The causes of delicate differences are analyzed after representing the sample feature. The copper film may be oxidized during the laser ablation process. The thickness of the copper film on the metasurface is asymmetrical because of the micromachining tolerance of the magnetron sputtering machine. Slight deviations between the structure size of the prepared sample and the physical dimensions of the simulated one are inevitable on account of the precision of the laser burning equipment.

## 4. Conclusions

A polarization sensitive MMA was first employed to design a PD and the PD absorptivity is first used to reflect and describe the polarization of incident waves in this paper. The sandwich structures of the presented PD composed of 0.1 μm periodic “I” shaped patches on the metasurface, a dielectric of 200 μm FR-4 on the interlayer, a 0.3 μm copper film on the substrate are uncomplicated, and the preparation technology is fully developed. The study of the influences on absorptivity of the incidence angles, polarization angles, and materials confirmed that the designed and prepared PD with absorbing responses located in the radar bands possesses excellent characteristics such as polarization sensitivity at all incidence angles and an ultrathin structure which are the basic elements for polarization detection. The sample prepared by laser ablation on the FR-4 board coated with copper on a double plane is executed with scattering parameter measurement in a microwave chamber. Compared with simulated results, the tested absorptivity was slightly decreased because of the fabricauion tolerance. Multiband and broadband PD in the desired resonance bands can be designed conveniently by adjusting the structure and size of resonators. The presented PD, which greatly enriches the metamaterial applications in polarized glasses, thermal images, stealth materials, communication and electromagnetic devices, should be easy to mass produce and market because of its strong detectability, ultrathin thickness, low cost, simple structure, and convenient manufacturing process.

## Figures and Tables

**Table 1:** Structure parameters.

Parameters	Value (μm)	Description
p	4000	periodic array length
h_s_	0.3	substrate thickness
h_d_	200	dielectric thickness
h_r_	0.1	metasurface thickness
l_1_	1200	“I” dimension
l_2_	1000	“I” dimension
l_3_	1000	“I” dimension
l_4_	100	“I” dimension

**Table 2:** Polarization extracted from resonance frequency and absorptivity.

Resonance Frequency (GHz)	Absorptivity	Polarization
36.9	0.94679	TE, θ = 0°, ϕ = 0°
16.055, 36.9	0.33029, 0.84501	TE, θ = 0°, ϕ = 45°
16.055	0.59401	TE, θ = 0°, ϕ = 90°
16.055	0.59692	TM, θ = 0°, ϕ = 0°
16.055, 36.9	0.33393, 0.84652	TM, θ = 0°, ϕ = 45°
36.9	0.95156	TM, θ = 0°, ϕ = 90°
36.9	0.99802	TE, θ = 40°, ϕ = 0°
16.055, 36.9	0.25826, 0.71298	TE, θ = 40°, ϕ = 45°
16.055	0.48603	TE, θ = 40°, ϕ = 90°
16.055, 41.41	0.6038, 0.42432	TM, θ = 40°, ϕ = 0°
16.055, 36.9, 41.355	0.34933, 0.87621, 0.31582	TM, θ = 40°, ϕ = 45°
36.845	0.96439	TM, θ = 40°, ϕ = 90°
36.845	0.5875	TE, θ = 80°, ϕ = 0°
16.055, 37.175	0.055418, 0.16297	TE, θ = 80°, ϕ = 45°
16.055, 43.775	0.13775, 0.21166	TE, θ = 80°, ϕ = 90°
16.11, 41.3, 43.17	0.96058, 0.95735, 0.58955	TM, θ = 80°, ϕ = 0°
16.055, 37.175, 41.63	0.73587, 0.96814, 0.87381	TM, θ = 80°, ϕ = 45°
37.065, 41.575, 43.72	0.74289, 0.85951, 0.76819	TM, θ = 80°, ϕ = 90°

## Abbreviations

The following abbreviations are used in this manuscript:

PD	Polarization Detector
MMA	Metamaterial Absorber
FIT	Finite Integration Technology
LIGO	Laser Interferometer Gravitational Wave Observatory
RGWs	Relic Gravitational Waves
CFRPs	Carbon Fiber Reinforced Plastics
LHMs	Left-Handed Materials
TEM	Transverse Electromagnetic
TE	Transverse Electric
TM	Transverse Magnetic
FSS	Frequency Selective Surface
FWHM	Full Width at Half Maximum

## References

[1] Leszek R.J., Pawel M. Fiber-optic interferometric polarization analyzer. Proceedings of the IEEE Instrumentation and Measurement Technology Conference.

[2] Peek T.H. (1971). Dynamic polarization detection. Opt. Commun..

[3] Azzam R.M. (1980). Longitudinal polarization-dependent coupling of light from an optical fiber to a side-bonded planar proximity detector: Application to integrated azimuthally distributed multidetector photopolarimeters. IEEE Photonic Technol. Lett..

[4] Azzam R.M. (1982). Division-of-amplitude photopolarimeter (DOAP) for the simultaneous measurement of all four stokes parameters of light. Opt. Acta.

[5] Azzam R.M., Bashara N.M. (1987). Ellipsometry and Polarized Light.

[6] Lu S.L., Li G.H. (1999). Make use of stokes son space to introduce Poincare sphere. J. Qufu Norm. Univ..

[7] Tong M.L., Zhang Y. (2008). Detecting very-high-frequency relic gravitational waves by electromagnetic wave polarizations in a waveguide. Chin. J. Astron. Astrophys..

[8] Miao H.X., Zhang Y. (2007). Analytic spectrum of relic gravitational waves modified by neutrino free streaming and dark energy. Phys. Rev. D.

[9] Grimberg R., Tian G.Y. (2012). High-frequency electromagnetic non-destructive evaluation for high spatial resolution, using metamaterials. Proc. R. Soc. A.

[10] Grimberg R. (2011). Electromagnetic nondestructive evaluation: Present and future. J. Mech. Eng..

[11] Grimberg R., Savin A., Steigmann R., Serghiac B., Bruma A. (2011). Electromagnetic non-destructive evaluation using metamaterials. Insight.

[12] Vesselago V.G. (1968). The electrodynamics of substances with simultaneously negative values of permittivity and permeability. Sov. Phys. Usp..

[13] Shelby R.A., Smith D.R., Schult S. (2001). Experimental verification of a negative index of refraction. Science.

[14] Pendry J.B., Smith D.R. (2004). Reversing light with negative refraction. Phys. Today.

[15] Luo C.Y., Ibanescu M., Johnson S.G., Joannopoulos J.D. (2003). Cherenkov radiation in photonic crystals. Semin. Dial..

[16] Seddon N., Bearpark T. (2003). Observation of the inverse doppler effect. Science.

[17] Berman P.R. (2002). Goos-hanchen shift in negatively refractive media. Phys. Rev. E.

[18] Alibakhshi K.M., Naser M.M., Virdee B.S., Andújar A., Anguera J. (2015). Compact antenna based on a composite right/left-handed transmission line. Microw. Opt. Technol. Lett..

[19] Schuring D., Mock J.J., Justice B.J. (2006). Metamaterial electromagnet cloak at microwave frequencies. Science.

[20] Pendry J.B. (2000). Negative refraction and the perfect lens. Phys. Rev. Lett..

[21] Martín F., Bonache J. (2014). Application of RF-MEMS-based split ring resonators (SRRs) to the implementation of reconfigurable stopband filters: A review. Sensors.

[22] Zhang Y., Feng Y.J., Zhu B., Zhao J.M., Jiang T. (2014). Graphene based tunable metamaterial absorber and polarization modulation in terahertz frequency. Opt. Exp..

[23] Zou T.B., Hu F.R., Xiao J., Zhang L.H., Liu F., Chen T., Niu J.H., Xiong X.M. (2014). Design of a polarization-insensitive and broadband terahertz absorber using metamaterials. Acta Phys. Sin..

[24] Savin A., Steigmann R., Bruma A., Šturm R. (2015). An electromagnetic sensor with a metamaterial lens for nondestructive evaluation of composite materials. Sensors.

[25] Tuong P.V., Park J.W., Lam V.D., Jang W.H., Nikitov S.A., Lee Y.P. (2013). Dielectric and ohmic losses in perfectly absorbing metamaterials. Opt. Commun..

[26] Costa F., Monorchio A., Manara G. (2014). An overview of equivalent circuit modeling techniques of frequency selective surfaces and metasurfaces. Appl. Comput. Electromagn..

[27] Costa F., Genovesi S., Monorchio A., Manara G. (2013). A circuit-based model for the interpretation of perfect metamaterial absorbers. IEEE Trans. Antennas Propag..

[28] Costa F., Monorchio A., Manara G. (2010). Analysis and design of ultra thin electromagnetic absorbers comprising resistively loaded high impedance surfaces. IEEE Trans. Antennas Propag..

[29] Costa F., Monorchio A., Manara G. (2012). Efficient analysis of frequency-selective surfaces by a simple equivalent-circuit model. IEEE Trans. Antennas Propag..

[30] Landy N.I., Bingham C.M., Tyler T., Jokerst N., Smith D.R., Padilla W.J. (2009). Design, theory, and measurement of a polarization insensitive absorber for terahertz imaging. Phys. Rev. B.

[31] Ye Q.W., Liu Y., Lin H., Li M.H., Yang H.L. (2012). Multi-band metamaterial absorber made of multi-gap SRRs structure. Appl. Phys. A.

